# Reproducibility of radiomic features using network analysis and its application in Wasserstein *k*-means clustering

**DOI:** 10.1117/1.JMI.8.3.031904

**Published:** 2021-04-30

**Authors:** Jung Hun Oh, Aditya P. Apte, Evangelia Katsoulakis, Nadeem Riaz, Vaios Hatzoglou, Yao Yu, Usman Mahmood, Harini Veeraraghavan, Maryam Pouryahya, Aditi Iyer, Amita Shukla-Dave, Allen Tannenbaum, Nancy Y. Lee, Joseph O. Deasy

**Affiliations:** a Memorial Sloan Kettering Cancer Center, Department of Medical Physics, New York, United States; b Veterans Affairs, James A Haley, Department of Radiation Oncology, Tampa, Florida, United States; c Memorial Sloan Kettering Cancer Center, Department of Radiation Oncology, New York, United States; d Memorial Sloan Kettering Cancer Center, Department of Radiology, New York, United States; e Stony Brook University, Department of Computer Science, Stony Brook, New York, United States; f Stony Brook University, Department of Applied Mathematics and Statistics, Stony Brook, New York, United States

**Keywords:** radiomics, reproducibility, Wasserstein distance, network-based Wasserstein *k*-means clustering

## Abstract

**Purpose:** The goal of this study is to develop innovative methods for identifying radiomic features that are reproducible over varying image acquisition settings.

**Approach:** We propose a regularized partial correlation network to identify reliable and reproducible radiomic features. This approach was tested on two radiomic feature sets generated using two different reconstruction methods on computed tomography (CT) scans from a cohort of 47 lung cancer patients. The largest common network component between the two networks was tested on phantom data consisting of five cancer samples. To further investigate whether radiomic features found can identify phenotypes, we propose a k-means clustering algorithm coupled with the optimal mass transport theory. This approach following the regularized partial correlation network analysis was tested on CT scans from 77 head and neck squamous cell carcinoma (HNSCC) patients in the Cancer Imaging Archive (TCIA) and validated using an independent dataset.

**Results:** A set of common radiomic features was found in relatively large network components between the resultant two partial correlation networks resulting from a cohort of lung cancer patients. The reliability and reproducibility of those radiomic features were further validated on phantom data using the Wasserstein distance. Further analysis using the network-based Wasserstein k-means algorithm on the TCIA HNSCC data showed that the resulting clusters separate tumor subsites as well as HPV status, and this was validated on an independent dataset.

**Conclusion:** We showed that a network-based analysis enables identifying reproducible radiomic features and use of the selected set of features can enhance clustering results.

## 1 Introduction

Radiomics enables an in-depth measurement of tumor phenotypes by quantifying imaging signals from radiologic images that can reflect key information of signatures associated with patient outcomes.^1^^,^^2^ Recently, the close connection of radiomics with machine learning has accelerated the development of new imaging features and radiomic outcomes modeling, showing the potential of using radiomics to build predictive models of individual cancer outcomes.^3^^,^^4^ Despite such great progress in radiomics in recent years, the development of computational techniques to identify repeatable and reproducible radiomic features remains challenging and relatively unadvanced.^5^ This has led to the lack of success of many radiomic models in subsequent external validation on independent data, impairing the reliability of the models.^6^^,^^7^ One of the reasons for this is likely due to the susceptibility of radiomic features to image reconstruction and acquisition parameters.^8^^,^^9^ Since radiomic features are computed via multiple tasks including imaging acquisition, segmentation, and feature extraction, the selection of parameters present in each step may affect the stability of features computed.^10^ As such, prior to model building, the development of radiomic features with high repeatability and high reproducibility as well as the development of tools that can identify such features is more likely to be urgently needed in the field of radiomics.

In this study, for the identification of reliable and reproducible radiomic features, we propose a graph (network)-based computational method, consisting of a partial correlation network analysis and the graphical lasso (linear absolute shrinkage and selection operator). We demonstrate its potential to identify reproducible radiomic features, using computed tomography (CT) images in lung cancer patients and validating the findings on phantom data. We employ the $W_{1}$-Wasserstein distance (also known as Earth Mover’s distance: EMD) as a quantitative metric to assess the reproducibility of radiomic features. To investigate whether radiomic features found by the regularized partial correlation network analysis can identify phenotypes, we further propose a method, the network-based Wasserstein k-means (NWK) clustering algorithm, to identify subgroups of tumors, employing the Wasserstein distance as a cost function in the conventional k-means algorithm. The method is tested on CT images in head and neck squamous cell carcinoma (HNSCC) patients downloaded from The Cancer Imaging Archive (TCIA) and validated using an independent dataset.

The hypothesis behind the proposed methodology is that radiomic features that retain the strong correlation with other features in a network form after removing spurious or false positive connections are likely to be reliable and reproducible.

## 2 Methods

### 2.1 Radiomics on CT Scans in Lung Cancer Patients

This study was approved by the Internal Review Board (IRB). In total, 47 lung tumors were segmented on contrast-enhanced CT images of lung cancer patients that were scanned using a GE MEDICAL SYSTEMS scanner and reconstructed with standard and lung convolution kernels. Initially, lung tumors were segmented on scans belonging to the lung reconstruction and were copied over to scans belonging to the standard reconstruction. For each reconstruction method, a set of 132 radiomic features was extracted using the CERR radiomics toolbox.^11^ Extracted features are categorized into three groups: (1) first-order statistics, (2) shape-based features, and (3) higher order texture features including gray-level co-occurrence matrix (GLCM), gray-level run length matrix (GLRLM), gray-level size zone matrix (GLSZM), neighborhood gray tone difference matrix (NGTDM), and neighboring gray-level dependence matrix (NGLDM).

### 2.2 Phantom Data

A multimaterial 3D printer (PolyJet Objet 260 Connex 3, Stratasys, Eden Prairie, Minnesota) with Voxel Print software was used for the deposition of droplets of ultraviolet-curable photopolymer resins in a layer-by-layer manner.^12^^,^^13^ In this study, two base photopolymer resins were employed including TangoPlus (material A) and VeroWhite (material B), which range in attenuation of ∼65 Hounsfield unit (HU) to 125 HU at 120 kVp. The resolution of the printer is on the order of 48×84×30  μm. Because the resolution is finer than that of typical CT scanners used at our institution (0.625×0.625×0.625  mm),^12^ multiple resin droplets were mixed in a single voxel to reproduce the contrast differences of tumor-specific patterns observed on patient CT scans. To 3D print, the fine gradients of tumor intensity patterns, the Floyd Steinberg dithering algorithm was used.

As a first step to produce phantoms, two separate 3D prints were developed. The first 3D print was modeled after a single patient from the RIDER dataset that consisted of patients with nonsmall-cell lung carcinoma (NSCLC). It was printed to physically visualize the lung vasculature, extent of the tumor, and its structure. The second 3D print was designed to capture the morphologic appearance and different intensity patterns seen on CT scans for the single NSCLC and four pancreatic ductal adenocarcinoma tumors. Since the surrounding tissue influences the local resolution and noise properties of tumors, the lesions were immersed in a heterogeneous background that was modeled after patients with advanced stage hepatic cirrhosis. The area and number of slices of the final 3D print were dictated by the size of the largest tumor.

Prior to dithering, the HU values of each tumor and background were separately converted to double precision intensities within a range of 0 to 1. The decimal values in the new intensity range dictated the amount of photopolymer material that would be deposited within any given voxel. Then, each slice was supersampled to the resolution of the 3D printer using the Whittaker–Shannon (SINC) interpolation.

Lastly, each slice was dithered to yield a set of layered raster images using the Floyd–Steinberg dithering algorithm. A single raster layer encodes the spatial allocation of a resin material. Since the 3D printer is capable of depositing three different resin materials, three sets of raster files were generated. Within any raster layer, a value of 1 indicates the deposition of material A and a value of 0 indicates that no material is deposited. The first set of bitmaps was inverted to mix two materials within a single voxel such that a value of 0 in the first set of bitmaps (material A) has a value of 1 in the second set of bitmaps (material B). Since two materials with opposing densities can generate the desired pattern differences, the third set of bitmaps consisted of all zeros.

After 3D printing, the phantom was scanned sequentially 30 times with a typical abdominal CT protocol using the following parameters: 120 kVp, 280 mA, pitch of 0.984, reconstructed slice thickness of 5 mm, and a reconstruction interval of 5 mm. Images were reconstructed using the filtered backprojection algorithm. Two sets of 132 radiomic features were extracted using the CERR radiomics toolbox for the standard and lung reconstruction kernels.^11^

### 2.3 CT Scans in Head and Neck Cancer Patients

For further radiomic network analysis, pretreatment CT scans with IV contrast for HNSCC patients were downloaded from the TCIA.^14^ The data were previously used in our other study.^15^ Below we briefly introduce the preprocessing and radiomic feature extraction steps. In total, 188 available cases were imported into the Eclipse treatment planning system (Varian Medical System, Palo Alto, California) for segmentation. Before delineating lesions of interest (ROIs), samples that do not meet the inclusion criteria such as primary tumor size and image quality were excluded. For the evaluable scans that fulfill the inclusion criteria, the primary tumor on CT scans was manually delineated by a radiation oncologist and the delineation was independently confirmed by a neuroradiologist. The presence of CT artifacts was further assessed within the primary tumor. Slices with streak artifacts were not delineated and excluded from the analysis. If the proportion of slices with streak artifacts in each tumor was larger than 50% of the number of slices, the case was excluded from the study.^16^ This quality test resulted in a set of 77 cases with 28 laryngeal, 11 oropharyngeal, and 38 oral cavity tumors. There were 13 HPV-positive and 64 HPV-negative tumors.^17^

Radiomic features were extracted using the CERR radiomics toolbox on resampled scans at the resolution of 0.6×0.6×3.5  mm.^11^ Due to the effect of subsampling slices, which is caused by dental artifacts, two-dimensional (2D) radiomic features were extracted: 104 radiomic features including first-order statistics and higher order texture features were computed from artifact-free slices.

Further feature stability and volume-independent tests were performed. For each scan, 100 independent datasets were generated, each of which consisted of 75% of the artifact-free slices and for each dataset 104 radiomic features were recomputed. Features with a median coefficient of variation >0.1 across all samples were removed. Features with high correlations with tumor volume (Spearman’s correlation coefficient>0.4) were also removed. These two tests resulted in 67 radiomic features that were used for subsequent analyses.

The radiomic analysis results on the TCIA data were validated using an independent dataset with 83 HNSCC patients treated at our institution. The validation cohort consisted of 1 laryngeal tumor, 31 oropharyngeal, and 51 oral cavity tumors, all with pretreatment CT scans with IV contrast. Among 32 patients with laryngeal and oropharyngeal tumors, 27 had HPV-positive and 5 had HPV-negative tumors. However, HPV status for 51 patients with oral cavity tumors was not available since HPV status on oral cavity tumors is not routinely obtained due to the low or rare prevalence of HPV positivity. We utilized the identical radiomic analysis pipeline (including segmentation, preprocessing, feature extraction, and network analysis) used in the analysis of the TCIA data.

### 2.4 Regularized Partial Correlation Network

For a network representation of radiomic features, we adopted a Gaussian graphical model of partial correlation coefficients in which each connection (link) in a network is represented as a partial correlation coefficient between two radiomic features (nodes) after conditioning on all other available features.^18^ This network may be intractably complex including many spurious connections, particularly for data with numerous features. To remove potential false positives and make the network representation more interpretable for a meaningful understanding of the data, a lasso-type regularization (graphical lasso) was employed in which a tuning parameter λ controls the sparsity of the network by shrinking partial correlation coefficients.^19^^,^^20^ More specifically, higher λ values make the network sparser whereas lower values make the network denser possibly with false-positive connections. To optimize the λ value that controls the tradeoff between keeping spurious connections and removing true connections in a network, we employed a method that optimizes the fit of the network to the data by minimizing the extended Bayesian information criterion (EBIC).^21^ Lastly, weak connections whose absolute partial correlation coefficients are <0.2 were further removed since those are likely to be false positives.

### 2.5 Wasserstein Distance

The optimal mass transport (OMT) is an active research area with an ever-increasing growth in its application in numerous fields, including medical imaging analysis, statistical physics, machine learning, and genomics.^22^^–^^25^ Here, we briefly describe the basic concepts underlying OMT. Let $P(R^{n})$ denote the space of probability measures on $R^{n}$ with finite second moments. Then, using the Kantorovich relaxed formulation^26^ of OMT, the Wasserstein distance (EMD) between $\mu ,\nu \in P(R^{n})$ is defined as follows:

$$W_{1}(\mu ,\nu )=\underset{\pi \in \Pi (\mu ,\nu )}{\inf}\int_{R^{n}\times R^{n}}\|x-y\|d\pi (x,y),$$ (1)

where Π(μ,ν) denotes the set of all joint probability measures π on $R^{n}\times R^{n}$ whose marginals are μ and ν. A computationally efficient reformulation of the Wasserstein distance can be defined such that the flux vector $m\in R^{n}$ is optimized in the following manner:

$$W_{1}(\mu ,\nu )=\underset{m}{\inf}\{\int_{R^{n}}\|m(x)\|dx|\mu -\nu -\nabla \cdot m=0\},$$ (2)

where ‖·‖ is the Euclidean norm.

An alternative graph-theoretic formulation of Eq. (2) to compute the Wasserstein distance on a network modeled as a weighted graph is defined as follows:

$$W_{1}(\mu ,\nu )=\underset{u}{\min}\{\overset{m}{\underset{i=1}{\sum }}\|u_{i}\||\mu -\nu -Du=0\},$$ (3)

where m is the number of edges in a network, $u_{i}$ are fluxes on the edges, and D is the incidence matrix with rows and columns indexed by the nodes and edges in the network such that every entry (i,k) is set to 1 if the node i is assigned to be the head of the edge k and is set to −1 if it is the tail of k. Using Eq. (3), we can compute the Wasserstein distance between two samples on a connected component of partial correlation network consisting of radiomic features. We used the CVX toolbox in the R language to optimize the OMT problem on a network.^27^

### 2.6 Network-Based Wasserstein k-Means Algorithm

The k-means algorithm is one of the most commonly used clustering algorithms that partition a given set of samples into c clusters, by minimizing the within-cluster sum of squares:

$$\mathrm{argmin}\overset{c}{\underset{i=1}{\sum }}\underset{x_{j}\in C_{i}}{\sum }{\left\|x_{j}-\mu_{i}\right\|}^{2},$$ (4)

where $\mu_{i}$ is the mean of samples in cluster $C_{i}$.^28^ Here, we propose a clustering method in which the cost function in the conventional k-means algorithm is replaced with the Wasserstein distance metric as shown in the following equation:

$$\mathrm{argmin}\overset{c}{\underset{i=1}{\sum }}\underset{x_{j}\in C_{i}}{\sum }W_{1}(x_{j},\mu_{i}).$$ (5)

In this approach, during the process of k-means clustering, distances from each sequentially updated centroid in c clusters to samples on a fixed optimized radiomic network are computed and clustering of the samples is performed such that the within-cluster sum of Wasserstein distance is minimized. More specifically, the Wasserstein distance computed using Eq. (3) on a given network is used in the k-means algorithm and then centroids are updated. Based on the updated centroids, the Wasserstein distance is computed again and the centroids are newly computed. This process is iterated until the centroids do not change or the number of iterations is reached to the predefined maximum number. The k-means algorithm coupled with the Wasserstein distance intuitively enables us to cluster samples in a network form of the given data. We call this method the NWK clustering algorithm.

## 3 Results

### 3.1 Regularized Partial Correlation Network

Two different reconstruction kernels (lung and standard) were applied to CT scans for a cohort of 47 lung cancer patients, generating a set of 132 radiomic features for each reconstruction method. For each set of features, a radiomic network was constructed using regularized partial correlation coefficients, i.e., an integrated method of partial correlation network analysis and graphical lasso. For the network optimization, the EBIC was employed with a default parameter setting. As a result, two different radiomic networks were constructed. To remove potential false-positive (or week) relationships between nodes (features) in the networks, connections with absolute partial correlation coefficients <0.2 were further removed. This may disconnect the network, creating more network components (islands). Hereafter, we define a component as a connected set of nodes in a network.

**Fig. 1:**
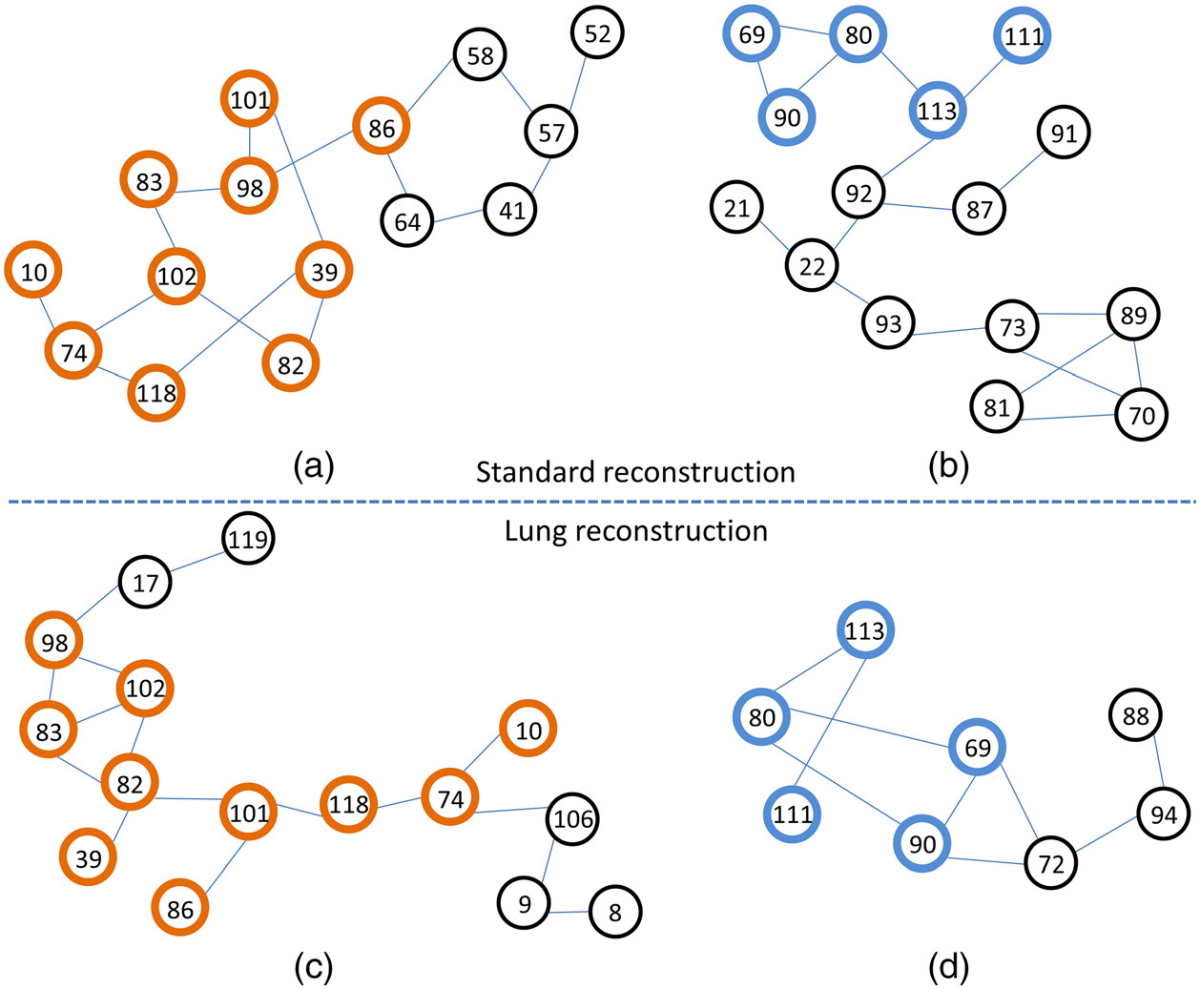
The two largest network components from (a) and (b) the standard reconstruction kernel and from (c) and (d) the lung reconstruction kernel. The thick circles with the same color indicate the common radiomic features between the two reconstruction methods. The numbers in the circles indicate the order of 132 features in our data.

Figure 1 shows the two largest network components for each reconstruction method with the number of links ≥9: Figs. 1(a) and 1(b) resulted from the standard reconstruction method whereas Figs. 1(c) and 1(d) resulted from the lung reconstruction method. In a comparison of Figs. 1(a) and 1(c), 10 radiomic features were common, including first-order statistics: entropy (10), shape: surface-to-volume ratio (39), GLRLM: run entropy (74), NGTDM: coarseness (82), contrast (83), strength (86), NGLDM: dependence count nonuniformity normalized (98), dependence count entropy (101), dependence count energy (102), and GLSZM: size zone entropy (118). In Figs. 1(b) and 1(d), five radiomic features were common including GLRLM: high gray-level run emphasis (69), short run high gray-level emphasis (80), NGLDM: high gray-level count emphasis (90), GLSZM: high gray-level zone emphasis (111), and small area high gray-level emphasis (113). In the similarity test of network components using the hypergeometric distribution, statistically significant p-values were obtained with $2.3\times 10^{-8}$ between Figs. 1(a) and 1(c) and $4.2\times 10^{-4}$ between Figs. 1(b) and 1(d), showing the strong reproducibility of these radiomic features between different reconstruction methods. The direct similarity of network topologies was not compared, but it is noted that most of the radiomic features preserve the connections with other features as shown in Figs. 1(a) versus 1(c) and Figs. 1(b) versus 1(d).

### 3.2 Validation on Phantom Data

**Fig. 2:**
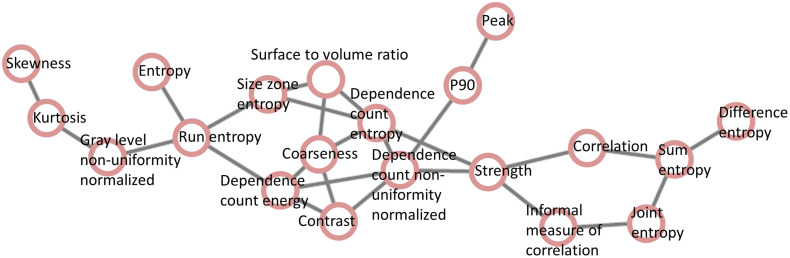
A combined network constructed by merging two network components, each of which is the largest network component in each reconstruction method, i.e., Figs. 1(a) and 1(c).

The reproducibility of those radiomic features identified using lung cancer data was further tested on phantom data. To do this, the two network components [Figs. 1(a) and 1(c)] each of which is the largest network component in each reconstruction method were combined, preserving the connections, which led to a connected network (Fig. 2) consisting of 20 radiomic features.

**Fig. 3:**
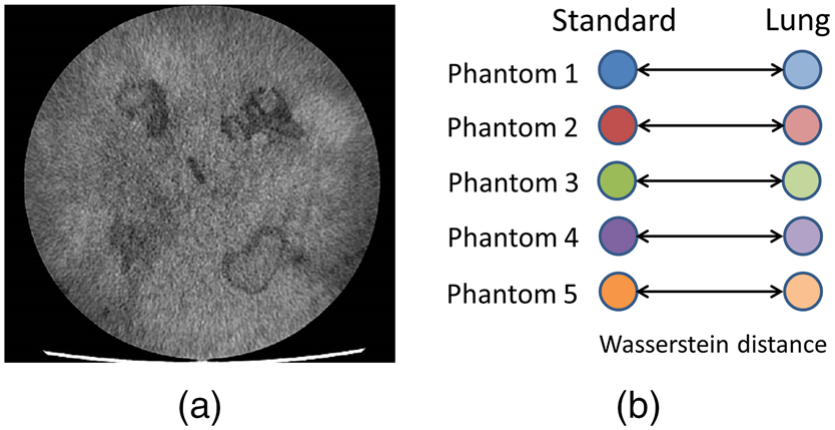
(a) An example of phantom slice used in this study. (b) For each phantom, the Wasserstein distance was computed between a set of features of standard reconstruction and a set of features of lung reconstruction on a network constructed using lung cancer data.

As previously described, five phantoms were generated in this study and for each phantom, two sets of radiomic features were extracted using the same two reconstruction kernels (standard and lung). Figure 3(a) shows a representative phantom slice used in this study. The same parameter setting in the CERR radiomics toolbox was used as in lung cancer analysis, generating 132 radiomic features for each set. Using Eq. (3), the Wasserstein distance was computed on the merged network (Fig. 2) between the two sets of 20 radiomic features for each phantom sample [Fig. 3(b)]. The average Wasserstein distance on the five phantoms was 0.21 (denoted as a reference for comparison with a simulation test shown in the following section) using the following equation:

$$\text{Average Wasserstein distance}=\frac{1}{5}\overset{5}{\underset{i=1}{\sum }}W_{1}({\text{Lung}}_{i},{\text{Standard}}_{i}).$$ (6)

**Fig. 4:**
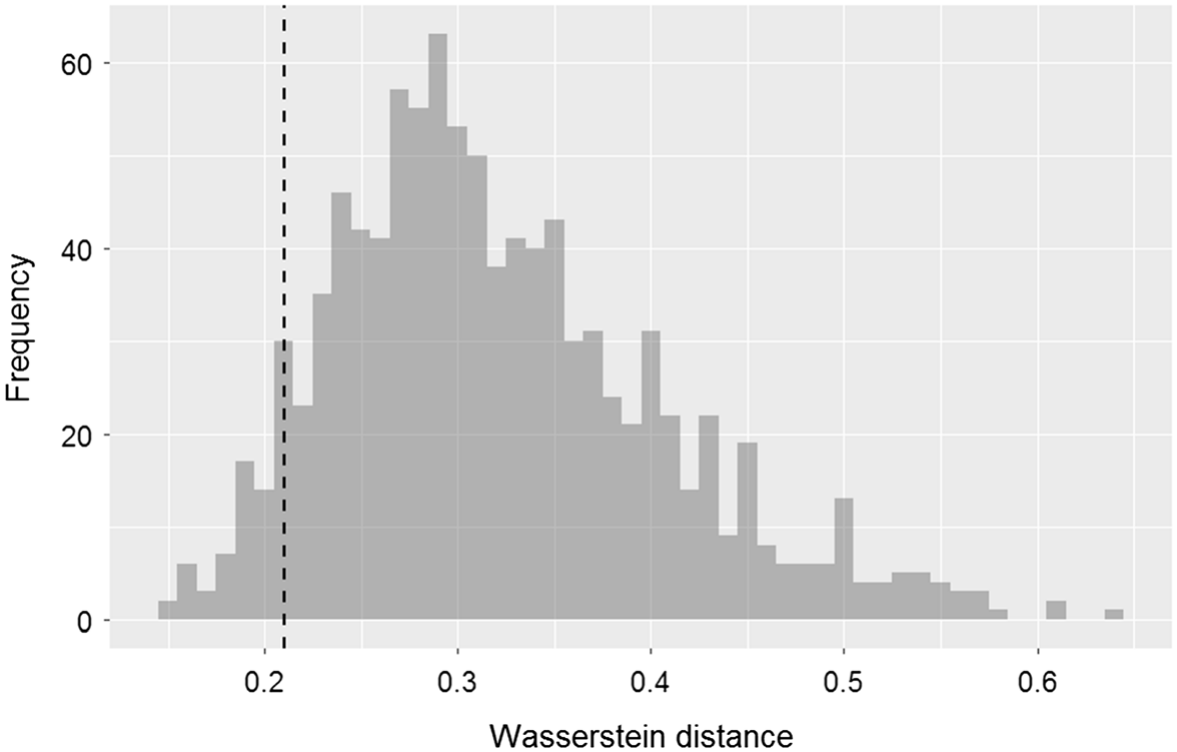
On the network shown in Fig. 2, a random simulation test was performed, by randomly selecting 20 features from the available 132 radiomic features and randomly assigning the 20 features to the 20 nodes in the network. This histogram shows the results of Wasserstein distance after 1000 iterations between a set of features of standard reconstruction and a set of features of lung reconstruction. The dotted vertical line indicates 0.21 that is an average Wasserstein distance of the five phantoms computed on the original network shown in Fig. 2.

It is likely that if the 20 radiomic features and their relationships (links) shown in Fig. 2 are reliable and stable, the Wasserstein distance computed using random radiomic features on the same network would be larger than 0.21 (reference). With this hypothesis, we randomly selected 20 features from the available 132 radiomic features and randomly assigned the 20 features to the 20 nodes in the network (Fig. 2) and then computed an average Wasserstein distance for five phantoms using Eq. (6). The whole process was repeated 1000 times, yielding 1000 average Wasserstein distance values. An overall average of the 1000 Wasserstein distance values was 0.32 and for only 49 times out of the 1000 simulation tests, the average Wasserstein distance was <0.21 (Fig. 4), implying the stability of the 20 radiomic features and their relationships in the network.

### 3.3 Network-Based Wasserstein k-Means Clustering

**Fig. 5:**
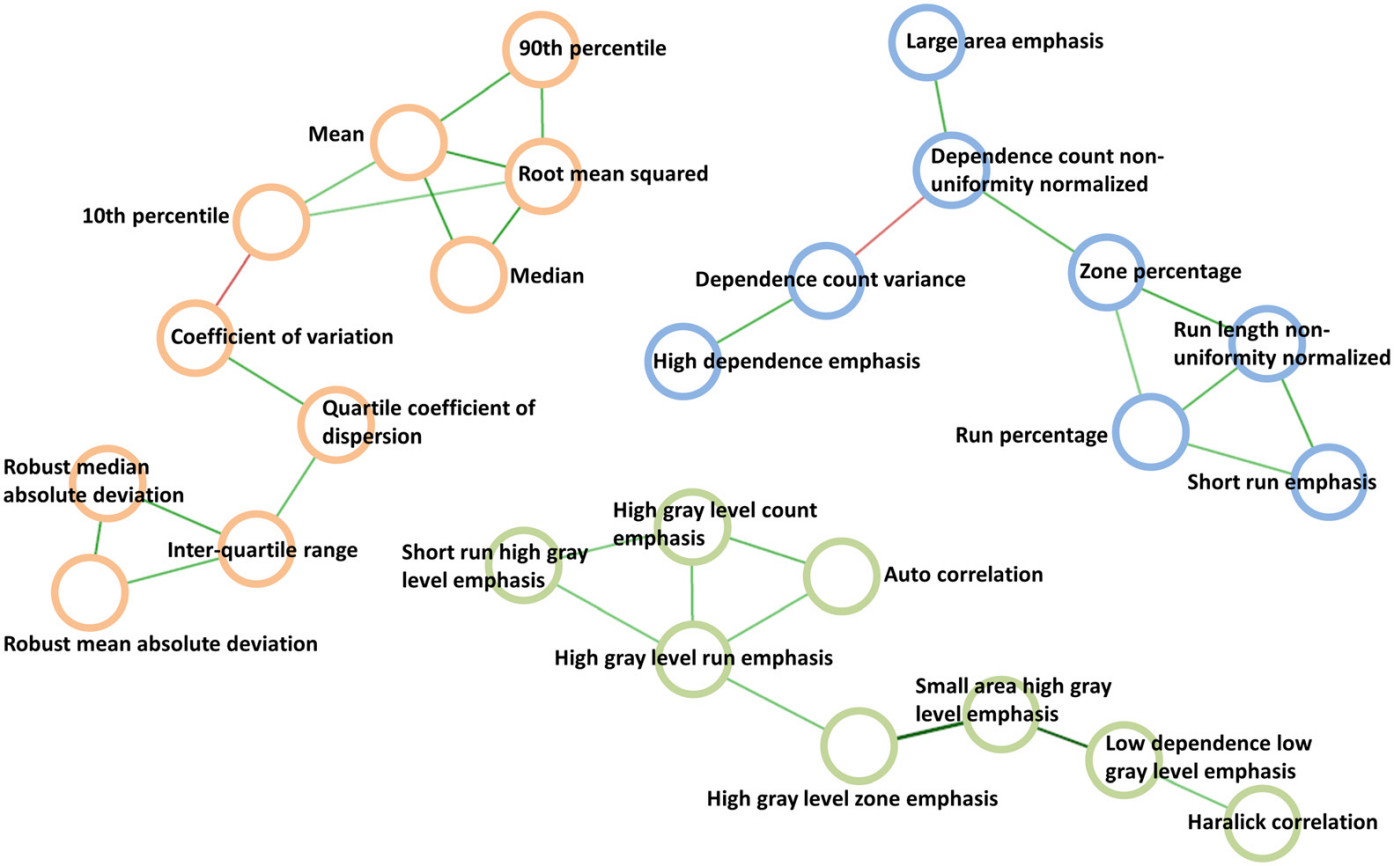
The three largest network components of partial correlation network that resulted from the TCIA head and neck cancer data.

Using the TCIA HNSCC data, consisting of 77 samples and 67 radiomic features for each sample, a regularized correlation network was constructed. After applying the EBIC and cutting the links with partial correlation coefficients <0.2, a final network was built. The three largest network components with the number of links ≥9 were chosen for further analysis, which consisted of 26 radiomic features (Fig. 5).

**Fig. 6:**
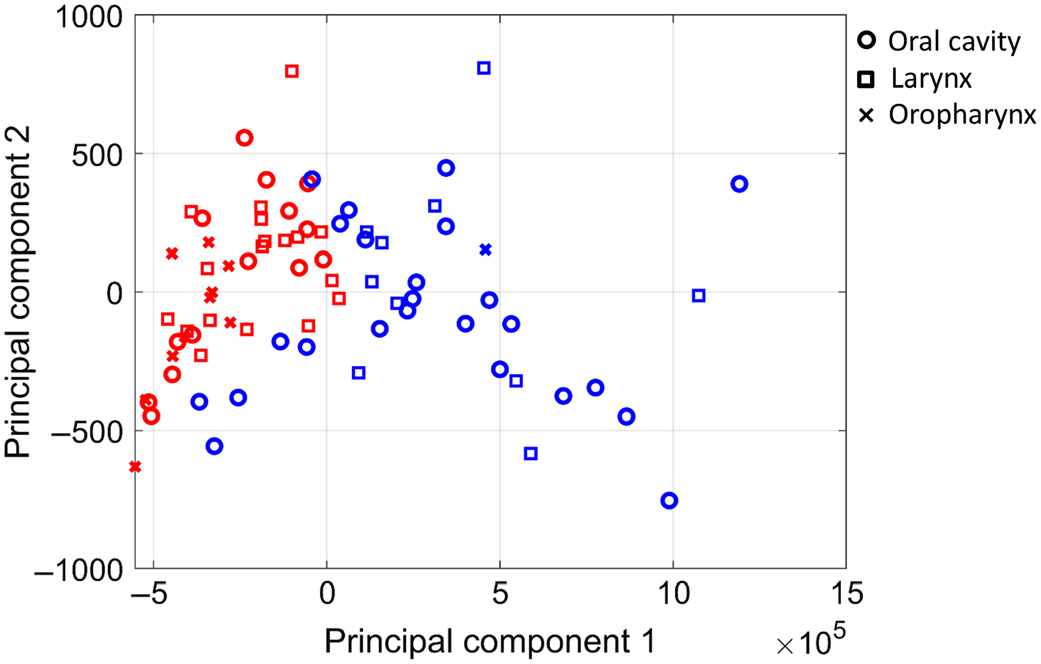
The NWK algorithm was performed on the TCIA head and neck cancer data. For visualization purpose, PCA was performed and the final clustering results were projected to the first two principal components. The blue and red colors indicate the two different clusters.

Based on the silhouette criterion, the optimal number of clusters was 2. Using the NWK algorithm with k=2, clustering was performed. During the k-means clustering process, the Wasserstein distance was computed for each network component and the three Wasserstein distance values were averaged. For the purpose of visualization, samples along with their clustering membership were represented on a low-dimensional space mapped from the 26 radiomic features, using principal component analysis (PCA). Figure 6 shows the clustering results visualized on the first two principal components.

A significant difference in the tumor subsites was found between the two clusters with extended Fisher’s exact test p=0.0027. One cluster (blue) in Fig. 6 had 1 oropharyngeal, 24 oral cavity, and 10 laryngeal tumors whereas the other cluster (red) had 10 oropharyngeal, 14 oral cavity, and 18 laryngeal tumors, and this cluster was significantly enriched for HPV-positive tumors with extended Fisher’s exact test p=0.030. These results were similar to what we previously reported in another study.^15^ It should be noted that we used a network as a basic representation of radiomic features in which subnetworks identified by the proposed method may indicate distinct physiological functions (which is out of scope in this study), and the clusters of samples that resulted from the NWK algorithm on the network may reflect phenotypes.

**Fig. 7:**
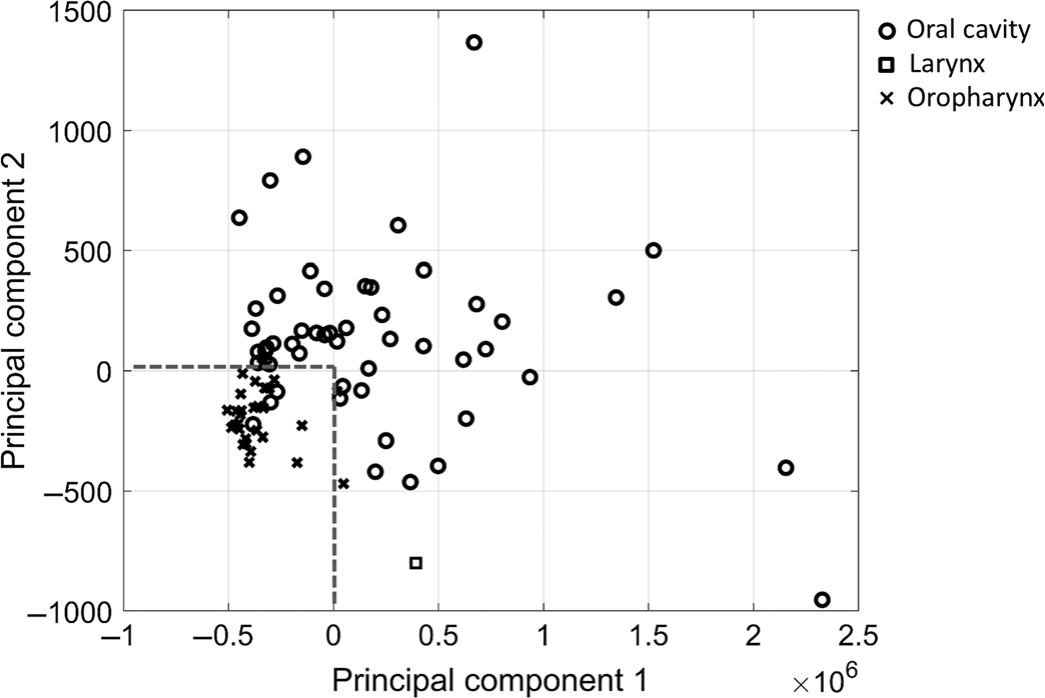
For validation, the 26 radiomic features were extracted from CT scans of 83 head and neck cancer patients treated at our institution. PCA was then carried out on the data. This scatter plot shows the projection results on the first two principal components.

For validation, the same 26 radiomic features were extracted from CT scans of 83 HNSCC patients treated at our institution and PCA was performed on the 26 features. Figure 7 shows the projection results on the first two principal components. Similarly, oropharyngeal tumors were clustered and separated from oral cavity tumors. Using a classifier with the dotted line boundary (both principal components have 0), the accuracy of classifying oropharyngeal tumors was 87.1% (27/31) and the accuracy of classifying oral cavity tumors was 94.1% (48/51). When all 67 radiomic features were used in PCA, the use of the same boundary on the first two principal components achieved an accuracy of 67.7% (21/31) and 90.2% (46/51) in classifying oropharyngeal and oral cavity tumors, respectively.

## 4 Discussion

Radiomics has shown great promise as a powerful tool in quantitatively characterizing tumor phenotypes and in improving predictive power of clinical outcomes modeling, particularly in conjunction with machine learning techniques. While over the last few years a number of new radiomic features have been developed and applied to outcomes modeling, the investigation into the reproducibility of such features still remains immature despite its importance for identifying reproducible and stable radiomic features, and thereby building reliable predictive models.

The use of unstable features in predictive modeling can lead to failure in validating the models on independent data. Virginia et al.^6^ reported a significant association between survival in NSCLC patients and primary mass entropy on CT scans using a training set, but this finding was not validated on a test set. Dissaux et al. investigated the capability of radiomic features extracted F18-FDG PET/CT to predict local recurrence in early-stage NSCLC patients treated with stereotactic radiotherapy. For the two multivariate models that achieved significant predictive power on a training set, a model using two PET radiomic features with information correlation 2 (IC2) from GLCM and texture strength from NGTDM remained statistically significant on a test set, whereas the other model using IC2 from PET and flatness from CT failed to reach statistical significance.^7^

As observed in our study, a partial correlation network analysis in connection with the graphical lasso showed the capability to identify reproducible radiomic features. The hypothesis underpinning this idea is that radiomic features that retain strong correlation with other features in a network formed after removing spurious or false-positive connections are likely to be reliable and reproducible. More specifically, the process to identify stable radiomic features consists of three steps, taking into account the connectivity among features in a network representation: (1) the correlation between two features is assessed, conditioning on all other available features; (2) spurious and potential false-positive connections are removed from the network using graphical lasso; and (3) weak connections are further removed. As a result, radiomic features identified through this filtering process are likely to be stable and reproducible across reconstruction methods.

Very few studies have been conducted for imaging analysis in a network form of radiomic features. Recently, a study was performed in a network analysis framework, using imaging features extracted from magnetic resonance images in intracranial ependymoma, and showed that subnetworks clearly separated tumor and healthy tissues, and even reflected tissue heterogeneity inside the tumor.^29^ However, to our knowledge, the present study is the first to exploit network analysis for identifying reproducible radiomic features.

We demonstrated the potential of network-based approaches to identify reproducible radiomic features on data obtained using two different reconstruction kernels on lung cancer CT scans. In a comparison of the largest network components between the two reconstruction methods, statistically significant p-values in terms of the common number of radiomic features were obtained with $2.3\times 10^{-8}$ between Figs. 1(a) and 1(c) and $4.2\times 10^{-4}$ between Figs. 1(b) and 1(d). Interestingly, it was found that most of the radiomic features preserved the relationships (links in the network) with other features between different reconstruction settings.

We further validated the reproducibility of those radiomic features on phantom data, employing the OMT metric, $W_{1}$-Wasserstein distance (EMD), as a quantitative metric. To further investigate whether radiomic features found by the regularized partial correlation network analysis can identify phenotypes, we proposed an NWK clustering method. This approach was applied to radiomic features from the TCIA HNSCC data after the regularized partial correlation analysis, resulting in two subgroups of tumors that significantly separated tumor sites and HPV status. On an independent dataset, a silhouette test resulted in a six-cluster solution. Due to its large number compared to the two clusters in the TCIA HNSCC data, we only assessed the capability of 26 radiomic features found in the TCIA HNSCC data to separate tumor sites on a low-dimensional space after the PCA projection. Its accuracy of classification was much better than that using all radiomic features, implying the reliability of the subset of features. Due to the lack of information on HPV status, the separation of HPV-positive tumors from HPV-negative tumors was not assessed.

In this study, radiomic features were extracted from 3D volume for lung cancer and phantom data whereas for the TCIA HNSCC CT scans, radiomic features were extracted from 2D slices due to CT scans with poor quality caused by dental artifacts. A recent study showed that there was no significant difference in the predictive power of positive and negative axillary lymph node status between 2D and 3D analysis, but further research is needed.^30^

In summary, we have adopted and developed network-based methods including regularized partial correlation analysis, the Wasserstein distance on a network, and the k-means algorithm coupled with the Wasserstein distance, which have the potential to identify reproducible radiomic features and their relationships in a network form as well as perform clustering of samples. Further, the proposed approach has an advantage compared to traditional statistical approaches that assess individual feature distributions in case that the number of feature distributions is limited; in this study, there are only two reconstruction datasets in the lung cancer cohort.

A major limitation of this study lies in the small sample size, in particular, in the size of phantom data. We plan to produce more phantoms and analyze additional reconstruction methods for further reproducibility test of radiomic features and its results will be compared with those in large real patients’ data. It should be noted that our approach is fully unsupervised learning without overfitting in the analysis. We also plan to develop a supervised method using the concept of Wasserstein distance, which enables us to build an individualized predictive model of outcomes.

## 5 Conclusion

In this study, we showed the potential of using a network-based approach to identify radiomic features that are reproducible over different image reconstruction methods. This was tested on CT scans in cancer patients and phantoms using the Wasserstein distance metric designed to compute the dissimilarity of samples on a network. We further proposed an NWK algorithm to cluster samples using the Wasserstein distance metric as a cost function. The clustering results on the TCIA data were validated using independent data. Applying the OMT coupled with the network analysis to radiomics could provide a powerful tool to identify reproducible radiomic features as well as develop reliable prediction models.
